# Evaluation and comparison of the sensitivity of three commercial RT-qPCR kits used for the detection of SARS-CoV-2 in Santiago, Chile

**DOI:** 10.3389/fpubh.2022.1010336

**Published:** 2022-11-28

**Authors:** Roberto Luraschi, Álvaro Santibáñez, Carlos Barrera-Avalos, Eva Vallejos-Vidal, Carlos Mateluna-Flores, Javiera Alarcón, Javiera Cayunao, Andrea Mella-Torres, Felipe Hernández, Ailen Inostroza-Molina, Daniel Valdés, Mónica Imarai, Claudio Acuña-Castillo, Felipe E. Reyes-López, Ana María Sandino

**Affiliations:** ^1^Centro de Biotecnología Acuícola, Facultad de Química y Biología, Universidad de Santiago de Chile, Santiago, Chile; ^2^Centro de Nanociencia y Nanotecnología CEDENNA, Universidad de Santiago de Chile, Santiago, Chile; ^3^Núcleo de Investigación Aplicada en Ciencias Veterinarias y Agronómicas, Facultad de Medicina Veterinaria y Agronomía, Universidad de Las Américas, Santiago, Chile; ^4^Departamento de Biología, Facultad de Química y Biología, Universidad de Santiago de Chile, Santiago, Chile

**Keywords:** SARS-CoV-2 detection, RT-qPCR, COVID-19, genomic surveillance, variants, diagnostic sensitivity

## Abstract

**Introduction:**

The COVID-19 pandemic is still in force, causing global public health challenges and threats. Although vaccination and herd immunity have proven to be the most efficient way to control the pandemic, massive and early testing of patients using the RT-qPCR technique is crucial for constant genomic surveillance. The appearance of variants of SARS-CoV-2 with new mutations can reduce the efficiency of diagnostic detection. In this sense, several commercial RT-qPCR kits have been the target of extensive analysis because low assay performance could lead to false-negative diagnoses.

**Methods:**

In this study, we evaluated the performance of three commercial RT-qPCR kits; Thermo Fisher (TaqMan 2019-nCoV Assay Kit v1), BGI and Roche (LightCycler^®^ Multiplex RNA Virus Master) used for the diagnosis of COVID-19 throughout the pandemic in Santiago de Chile.

**Results:**

Under our best assay conditions, we found significant differences in Cq amplification values for control and viral probes, against the same nasopharyngeal swab samples (NPSs). In addition, in some cases, the sensitivity of the RT-qPCR kits decreased against viral variants.

**Conclusion:**

Our study suggests evaluating the RT-qPCR kits used to detect SARS-CoV-2 because variants such as Omicron, which has several mutations, can compromise their detection and underestimate viral circulation.

## Introduction

Coronavirus disease 2019 (COVID-19) is caused by severe acute respiratory syndrome coronavirus 2 (SARS-CoV-2). Since it was declared a pandemic by the World Health Organization (WHO) on March 11, 2020 (1), it has generated a challenge for health authorities and systems today. Mass vaccination is the best way to control viral infection and prevent severe illness (2), however, the appearance of new viral variants and the decline of immunity from vaccines (3–5) forces the health authorities to constantly monitor and test the population on a massive scale. Real-time reverse transcription-polymerase chain reaction (RT-PCR) is the gold-standard molecular diagnostic tool for detecting SARS-CoV-2. Consequently, RT-PCR is the most effective strategy for testing, tracing, and isolating positive cases today, over rapid antigen tests (6). For this reason, the different commercial RT-qPCR kits have been the subject of extensive performance analysis since a low performance or sensitivity, could underestimate viral circulation in the community (7–9), even of variants of SARS-CoV-2 (10). These analyzes are necessary, especially in South American countries, where the low quality of some supplied RT-qPCR kits has been denounced (11).

The diagnosis of COVID-19 has been based on detecting a series of target genes of SARS-CoV-2. For example, screening includes viral RNA for structural proteins such as envelope (E), nucleocapsid (N), and spike (S) and open reading frame 1ab (ORF1ab), which encode nonstructural proteins, such as dependent polymerase RNA (RdRp) (12). In this line, a sensitivity range of 96%-100% has been reported detecting the viral gene ORF1ab, RdRP, protein N, and S (8, 13). On the other hand, sensitivity differences of up to 25% have been observed in RT-qPCR kits detecting the same viral genes (14), suggesting overall-quality differences between different manufacturers.

Despite the existing reports on the performance of RT-qPCR kits on the market for the diagnosis of COVID-19, no studies are comparing the detection sensitivity of RT-qPCR kits used in Chile during the pandemic, which was considered the country that best managed the pandemic in Latin America (15). In this study, we evaluated the performance of three commercial RT-qPCR kits for the diagnosis of SARS-CoV-2, massively used to control the pandemic in Chile, including the TaqMan 2019-nCoV Assay Kit v1 (Thermo Fisher), the real-time fluorescent RT-PCR kit for detect SARS-CoV-2 (BGI) and LightCycler^®^ Multiplex RNA Virus Master (Roche). We report differences in Cq values for the internal control (IC) and the viral probes. In addition, we noted differences in sensitivity at samples with Cq > 30 and the detection of variants, such as Omicron. Our results highlight the relevance of analyzing the performance of RT-qPCR kits for diagnosing SARS-CoV-2, especially in the face of new viral variants currently circulating.

## Materials and methods

### Samples and total RNA extraction

Nasopharyngeal samples (NPSs) of clinical patients included in this study were collected by the Primary Care Centers and the Hospitals that belong to the Central Metropolitan Health Service (SSMC by its acronym in Spanish) in Santiago of Chile. The wild-type samples correspond to NPSs diagnosed in June 2020 at the beginning of the pandemic in Chile. The samples corresponding to the Gamma and Omicron variants of SARS-CoV-2 were diagnosed in April 2021 and April-May 2022, respectively. The NPSs were taken, preserved, and transported using the Genosur sampling and transport kit (catalog number: DM0001VR; Genosur LLC, NW). All samples arrived at the laboratory within the first 24 h after sampling. These samples were processed in the Clinical Laboratory of Virology (Universidad de Santiago de Chile, USACH). Total RNA extraction was carried out using the Total RNA Purification 96-well kit (Cat. No. 24380, Norgen Biotek Corp; Canada), following the manufacturer's instructions using as starting material the constant volume of 250 μl of NPSs as previously described (16).

### SARS-CoV-2 detection by RT-qPCR

The detection of viral SARS-CoV-2 was carried out using the ORF1ab probe (TaqMan™ 2019nCoV Assay Kit v1, Thermo Fisher Scientific, Cat. No. A47532) using a one-step strategy. Positive commercial control probes for ORF1ab and RNase P were included and assessed individually in the 96-well PCR plate. The polymerase from TaqMan™ Fast Virus 1-Step Master Mix (Applied Biosystems™, Cat. No. 44-444-36) was included in each reaction. Each reaction contained 5 μl of TaqMan™ Fast Virus 1-Step Master Mix 4X, 1 μl of ORF1ab assay 20X (FAM detector channel), 1 μl of RNase P assay 20X (HEX detector channel), 11 μl of nuclease-free water, and 2 μl of the extracted RNA sample. When the RT-qPCR reaction used 5 μl of extracted RNA as a template, 8 μl of nuclease-free water was dispensed (Table 1). The BGI kit detects viral SARS-CoV-2 using the ORF1ab probe (Real-Time Fluorescent RT-PCR Kit for detecting SARS-CoV-2, BGI Health (HK) Co. Ltd, China, Cat. No. MFG030010) using a one-step strategy. Positive commercial control probes for ORF1ab and β-actin were included and assessed individually in the 96-well PCR plate. The polymerase from BGI Reaction Mix (BGI Health (HK) Co. Ltd, China, Cat. No. MFG030010) was included in each reaction. Each reaction contained 18.5 μl of SARS-CoV-2 Reaction Mix (HEX detector channel to β-actin and FAM detector channel to ORF1ab), 1.5 μl SARS-CoV-2 Enzyme Mix, 8 μl of nuclease-free water, and 2 μl of the extracted RNA sample. When the RT-qPCR reaction used 10 μl of extracted RNA as a template, nuclease-free water was not dispensed in the reaction (Table 2). The LightCycler^®^ Multiplex RNA Virus Master kit (Roche) detects viral SARS-CoV-2 genome using the RdRp probe (LightMix^®^ Modular Wuhan CoV RdRP-gene. Cat. No. 53-0777-96) using a one-step strategy. A positive commercial control probe for RdRp (LightMix^®^ Modular Wuhan CoV RdRp-gene. Cat. No. 53-0777-96) was included and assessed individually in the 96-well PCR plate. As a reference commercial control, the RNase P probe (TaqMan™ 2019-nCoV Control Kit v1; Thermo Fisher Scientific, Cat. No. A47533) was included to ensure the presence of total RNA extracted from NPSs as a template. This decision was supported because the Roche RT-qPCR kit used the Equine Arteritis Virus (EAV) as an internal control for the extraction process but not a control of the total RNA extracted. The polymerase from RT-qPCR Reaction Mix 5x (The LightCycler^®^ Multiplex RNA Virus Master kit, Cat. No. 06754155001) was included in each reaction. Each reaction contained 0.5 μl of RdRp probe (FAM detector channel), 4 μl of RT-qPCR Reaction Mix 5X, 0.1 μL of RT Enzyme Solution 200X, 1 μL of RNase P probe, 12.4 μl of nuclease-free water, and 2 μl of the extracted RNA sample. When the reaction used 5 μl of the extracted RNA as a template, 9.4 μl of nuclease-free water was dispensed (Table 3). All the RT-qPCR reactions were performed on the Agilent AriaMx Real-Time PCR System (Agilent Technologies, Part. No. G8830A). Data were extracted using the Agilent AriaMx software. The thermal amplification conditions for three RT-qPCR kits include the reverse transcription at 50 °C for 10 min, RT inactivation and initial denaturation step at 95 °C for 30 s, followed by 45 cycles of denaturation step at 95 °C for 5 sec and, annealing and extension step at 60 °C for 30 sec (Table 4).

**Table 1:** Thermo Fisher RT-qPCR mix.

**Reagent**	**Vol (μl)**
TaqMan™ Fast Virus 1-Step Master Mix 4X	5
ORF1ab assay 20X	1
RNase P assay 20X	1
Nuclease-free water	11 (8)
RNA	2 (5)
Final volumen	20

**Table 2:** BGI mix RT-qPCR mix.

**Reagent**	**Vol (μl)**
SARS-CoV-2 Reaction Mix	18.5
SARS-CoV-2 Enzyme Mix	1.5
Nuclease-free water	8 (0)
RNA	2 (10)
Final volumen	30

**Table 3:** Roche RT-qPCR mix.

**Reagent**	**Vol (μl)**
RdRP probe	0.5
RT-qPCR Reaction Mix 5X	4
RT Enzyme Solution 200x	0.1
RNase P probe	1
Nuclease-free water	12.4 (9.4)
RNA	2 (5)
Final volumen	20

**Table 4:** RT-qPCR thermal protocol.

**Step**	**T (°C)**	**Thermo Fisher**	**BGI**	**Roche**	**Cycles**
Reverse transcription	50	5 min	5 min	10 min	1
RT inactivation / initial denaturation	95	20 seg	20 seg	30 seg	1
Denaturation	95	3 seg	3 seg	5 seg	45
Anneal / extention	60	30 seg	30 seg	30 seg	

### RT-qPCR efficiency, Cq cut-off and sensitivity

To establish PCR efficiency and the Cq cut-off for the internal control (IC) probes (RNase P for Thermo Fisher and Roche RT-qPCR kits; β-actin for BGI kit) and the viral probes (ORF1ab for Thermo Fisher and BGI kits; RdRp for Roche kit), we ran RT-qPCR reaction using serial dilutions. We used a reference pool made with nuclease-free water for the 10-fold serial dilutions from total RNA from NPSs with a Cq value of around 20 (chosen from positive samples on the same day). The reactions were carried out according to the specific conditions indicated by the manufacturer and described above. All the RT-qPCR reactions were performed on the Agilent AriaMx Real-Time PCR System. We determined the slope by linear regression in GraphPad Prism and defined the required levels for PCR efficiency (%Eff) and R-squared (R^2^) as>95% and >0.95, respectively. The primer efficiency was calculated according to the formula Efficiency % (E) = (10^(−1/Slope)^-1)·100 (17). From this analysis, the Cq cut-off of the probes of each RT-qPCR kit was determined, understood as the average of the Cq values of the last dilution that showed amplification. The linear regression of a standard curve made for each probe using 10-fold serial dilutions of the positive control of its respective RT-qPCR kit quantified the number of genome copies/μl detected by each RT-qPCR kit. The sensitivity of the three RT-qPCR kits evaluated was based on that described by Trevethan et al. (18), establishing the Thermo Fisher kit as the gold standard for identifying and diagnosing SARS-CoV-2.

### SARS-CoV-2 variants determination

Samples with Cq < 30 were analyzed for the detection of SARS-CoV-2 variants, as part of a national strategy to monitor the circulation of SARS-CoV-2. The positive samples detected in our laboratory (NPSs Cq < 30) were referred to the Public Assistance Emergency Hospital (HUAP). The “Allplex™ SARS-CoV-2 Variants I” (Cat. No. RV10286X) and “Allplex™ SARS-CoV-2 Variants II Assay” (Cat. No. RV10305X) RT-qPCR kits were used, according to the manufacturer's specifications. The results were then uploaded to the national COVID-19 results platform. The combination of mutations K417T, E484K, and N501Y indicated the presence of the Gamma variant, while mutations K417T, del69-70, and N501Y indicated the presence of the Omicron variant. Subvariants were not analyzed or classified.

### Ethics statement

All the experimental procedures included in this study was authorized by the Ethical Committee of the University of Santiago of Chile (No. 226/2021) and the Scientific Ethical Committee of the Central Metropolitan Health Service, Ministry of Health, Government of Chile (No. 370/2021), and following the Chilean law in force.

### Data representation and statistical analysis

The mean ± standard deviation (mean ± SD) for Cq and RFU results were represented in graphs. The Cq values are physically represented in the figures according to the relative abundance of amplicon detected, i.e., low Cq values (high amplicon abundance) are represented in a higher position than high Cq values (low amplicon abundance). A paired two-sided Student *T*-test was used to determine differences between the Cq and RFU values obtained by loading two different RNA-extracted volumes using the same RT-qPCR kit. A paired two-way ANOVA was used to determine differences between the Cq and RFU values obtained from the different SARS-CoV-2 RT-qPCR detection kits. A *p*-value of < 0.05 was considered statistically significant. GraphPad Prism 8 statistical software was used to analyze and plot the data obtained.

### Data availability statement

The data used to support the findings of this study can be released upon direct request to the corresponding author, who can be contacted *via* e-mail (felipe.reyes.l@usach.cl).

## Results

### Standardization of Thermo Fisher kit test conditions

**Figure 1:**
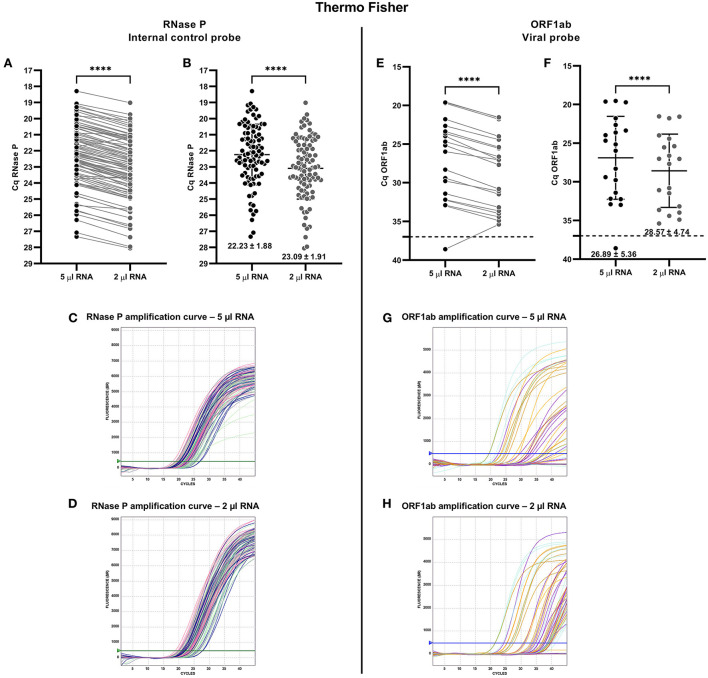
Comparative analysis for detecting SARS-CoV-2 from nasopharyngeal swab (NPS) samples using RNase P and ORF1ab gene probes from Thermo Fisher RT-qPCR kit. The comparison was made from the same NPS sample loading the recommended volume of extracted RNA (5 μl of total RNA, recommended by the manufacturer; black spots) and 2 μl of total RNA (gray spots). Each spot is an analyzed sample for each volume condition (5 μl; 2 μl). For graphs **(A,E)**, the line connecting the points shows the paired results obtained from the same sample analyzed by loading the two different volumes. Cq = 46 denotes no amplification (No Cq). **(A)** Paired quantification cycle (Cq) analysis for RNase P probe of each sample assessed. **(B)** Cq means value (mean ± SD) for the RNase P probe amplification. RNase P probe amplification curves loading **(C)** 5 μl and **(D)** 2 μl of total RNA. **(E)** Paired Cq analysis for ORF1ab probe of each sample assessed. **(F)** Cq means value (mean ± SD) for the ORF1ab probe amplification. The broken line indicates the Cq cut-off value (Cq 1≤ 37) recommended by the manufacturer. ORF1ab probe amplification curves loading **(G)** 5 μl and **(H)** 2 μl of total RNA. For statistical analysis, paired two-sided Student *T*-test was applied (*n* = 91 NPS blind selected samples, 20 positives for SARS-CoV-2). ^****^p < 0.0001.

For the standardization of the best conditions of the assay values for each RT-qPCR kit, NPSs samples corresponding to ancestral SARS-CoV-2 (wild type; collected in June 2020) were used. The analysis of the extracted NPSs samples with the Thermo Fisher RT-qPCR kit loading 5 μl (according to the manufacturer instructions) and 2 μl of total RNA revealed differences both in the quantification cycle (Cq) and in the relative fluorescence units (RFU) determined by the RNase P probe (IC probe) and ORF1ab viral probe (SARS-CoV-2 gene probe) amplification (Figure 1). From a global perspective, the 2 μl of total RNA template increased the Cq values for RNase P in most of the samples assessed compared to the 5 μl of total RNA template (Figure 1A). This first perception is reinforced when the mean ± standard deviation (mean ± SD) of the Cq values is plotted, showing a statistically lower mean Cq value for the 5 μl of total RNA template (22.23 ± 1.88) than 2 μl of template total RNA (23.09 ± 1.91) (Figure 1B).

On the other hand, the RFUs observed for the RNase P probe showed a substantial difference between both template volumes (Supplementary Figure 1A), showing a higher mean fluorescence for the 2 μl total RNA template (7686 ± 623.4) than 5 μl of total RNA template (5781 ± 698.3) (Supplementary Figure 1B).

Visualization of the amplification curves of the RNase P probe reinforces the idea of the difference observed between 2 μl and 5 μl of total RNA. Most of the samples showed curves with a sigmoid behavior, with a steeper slope and higher fluorescence for the 2 μl of NPSs RNA (Figure 1D) compared to the 5 μl of total template RNA (Figure 1C). When Thermo Fisher RT-qPCR evaluated the presence of SARS-CoV-2 genome loading 5 μl of total RNA extracted from NPS, 19 from 20 samples were diagnosed as COVID-19 positive, respectively (Figure 1E). In this sense, the sample with negative diagnoses showed a Cq value for ORF1ab probe of 38.59 when 5 μl of RNA was loaded (Figure 1F). From a global perspective, the 2 μl of total RNA template increased the Cq values for ORF1ab probe in almost all samples assessed, compared to the 5 μl of total RNA template, showing a Cq mean value of 28.57 ± 4.75 and 26.89 ± 5.36, respectively. However, by loading 2 μl of total RNA template instead of 5 μl, the diagnosis of false negative samples is avoided (Figure 1F).

Like RNase P probe amplification, most of the total RNA NPS-extracted samples registered a higher ORF1ab probe RFU for the 2 μl compared to the 5 μl of total RNA as template (Supplementary Figure 1C). Thus, the 2 μl of total RNA showed the RFU mean values of 4243 ± 521.6 in comparison with the 5 μl of total RNA that showed 3779 ± 1160 (Supplementary Figure 1D).

This effect on the RFU curves is seen in the ORF1ab probe amplification curves for 2 μl (Figure 1H), and 5 μl loaded total RNA (Figure 1G), reflecting higher amplification quality when loaded 2 μl of total RNA. In summary, the analysis of the performance of the Thermo Fisher RT-qPCR kit showed a higher quality of the SARS-CoV-2 detection assay by loading 2 μl of total RNA instead of the 5 μl recommended by the manufacturer.

### Standardization of BGI kit test conditions

**Figure 2:**
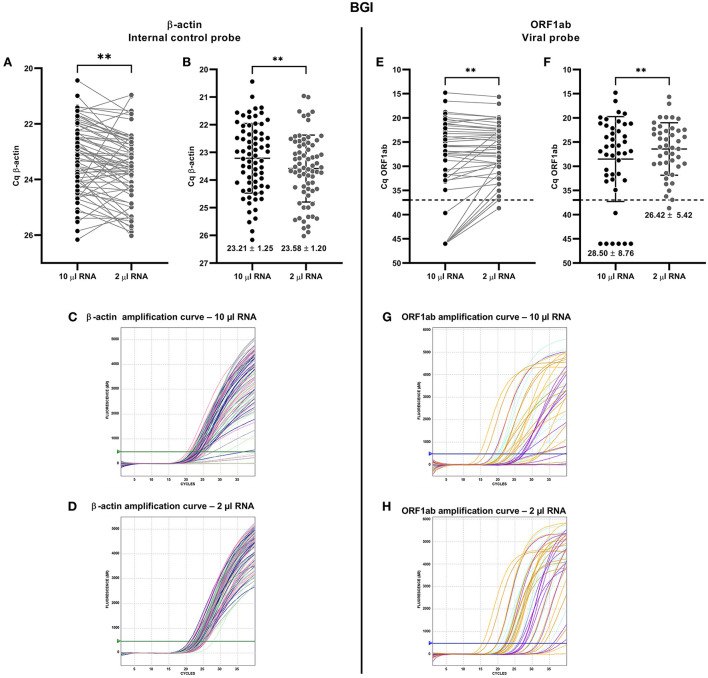
Comparative analysis for detecting SARS-CoV-2 from nasopharyngeal swab (NPS) samples using β-actin and ORF1ab gene from BGI RT-qPCR kit. The comparison was made from the same NPS sample loading the recommended volume of extracted RNA (10 μl of total RNA, recommended by the manufacturer; black spots) and 2 μl of total RNA (gray spots). Each spot is an analyzed sample for each volume condition (10 μl; 2 μl). For graphs **(A,E)**, the line connecting the points shows the paired results obtained from the same sample analyzed by loading the two different volumes. Cq = 46 denotes no amplification (No Cq). **(A)** Paired quantification cycle (Cq) analysis for the β-actin probe of each sample assessed. **(B)** Cq means value (mean ± SD) for the β-actin probe amplification. β-actin probe amplification curves loading **(C)** 10 μl and **(D)** 2 μl of total RNA. **(E)** Paired Cq analysis for ORF1ab probe of each sample assessed. **(F)** Cq means value (mean ± SD) for the ORF1ab probe amplification. The broken line indicates the Cq cut-off value (Cq ≤ 37) recommended by the manufacturer. ORF1ab amplification curves loading **(G)** 10 μl and **(H)** 2 μl of total RNA. For statistical analysis, paired two-sided Student *T*-test was applied (*n* = 70 NPS blind selected samples, 43 positives for SARS-CoV-2). **p < 0.01.

The BGI RT-qPCR kit also recorded differences in parameters between the volume recommended by the manufacturer (10 μl) and 2 μl of total RNA. β-actin amplification (IC probe) showed slightly lower Cq values for 10 μl total RNA compared to 2 μl template total RNA in most samples tested (Figure 2A). These data are supported by the mean ± SD of all samples tested, showing a decrease in the mean Cq value for the 10 μl total RNA template (23.21 ± 1.25) than the 2 μl of total RNA (23.58 ± 1.20) (Figure 2B).

On the other hand, the RFU was higher when a volume of 2 μl of total RNA was loaded, instead of 10 μl (Supplementary Figure 1E). This is confirmed by the higher RFU mean loading 2 μl (4242 ± 666.2) than the 10 μl of total RNA template (3718 ± 865.0) (Supplementary Figure 1F). When visualizing the amplification curves of the β-actin probe, most of the samples presented more resolved (sigmoid) curves when 2 μl of total RNA were loaded (Figure 2D) compared to when 10 μl of total RNA were loaded (Figure 2C), indicating a higher quality of assay and result when 2 μl of total RNA is loaded. Interestingly, at the time SARS-CoV-2 was evaluated in the total RNA NPS-extracted samples, very similar results were observed between the paired Cq values for 10 and 2 μl of total RNA in positive samples (Figure 2E). However, six samples diagnosed as COVID-19 negative with 10 μl of total RNA were determined as COVID-19 positive when 2 μl of total RNA were loaded (Cq_10μ*l*_ = 39.66 and Cq_2μ*l*_ =31.77; Cq_10μ*l*_ = No Cq and Cq_2μ*l*_ = 35.05; Cq_10μ*l*_ = No Cq and Cq_2μ*l*_ = 33.54; Cq_10μ*l*_ = No Cq and Cq_2μ*l*_ = 28.79, Cq_10μ*l*_ = No Cq and Cq_2μ*l*_ = 36.92, Cq_10μ*l*_ = No Cq and Cq_2μ*l*_ = 36.23) (Figure 2F). This result is evidenced by the slight lower Cq mean value for SARS-CoV-2 ORF1ab detection with 2 μl (26.42 ± 7.16) than 10 μl (28.50 ± 9.52) (Figure 2F).

As observed for the amplification of the β-actin probe, all the total RNA NPS-extracted samples registered a much higher fluorescence for the ORF1ab probe when 2 μl of total RNA was loaded (4357 ± 1239) compared to when 10 μl of total RNA was loaded (2872 ± 1727) (Supplementary Figures 1G,H). Amplification curves with 2 μl of RNA showed a better sigmoid pattern, and higher fluorescence (Figure 2H) compared to 10 μl of total RNA (Figure 2G), reflecting a higher quality of amplification and diagnosis. Thus, these results with the BGI RT-qPCR kit suggest a better detection of SARS-CoV-2 when loading 2 μl of total RNA instead of the 10 μl recommended by the manufacturer, being the condition chosen for the various subsequent analyses.

### Standardization of Roche kit test conditions

**Figure 3:**
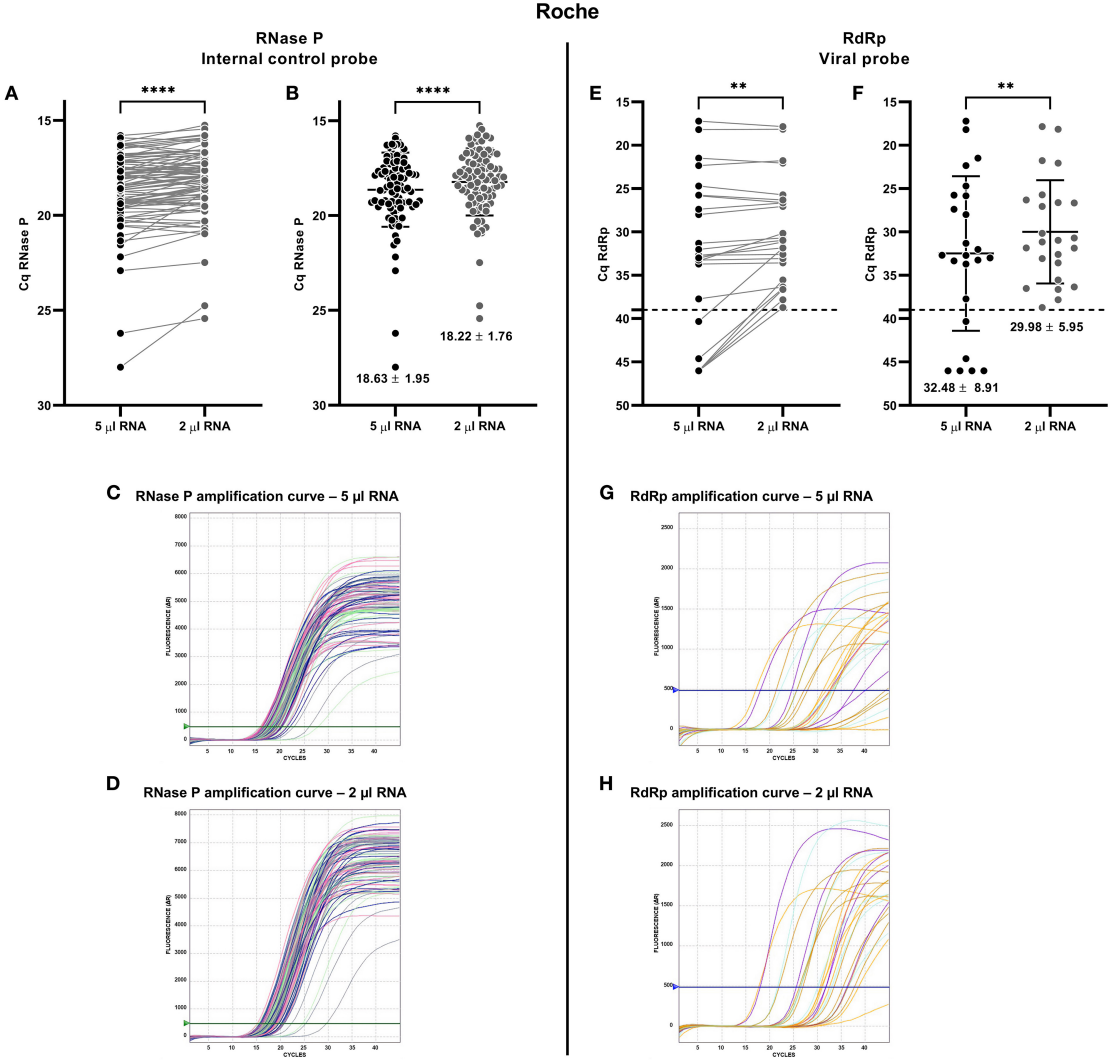
Comparative analysis for detecting SARS-CoV-2 from nasopharyngeal swab (NPS) samples using RNase P and RdRp gene probes from Roche RT-qPCR kit. The comparison was made from the same NPS sample loading the recommended volume of extracted RNA (5 μl of total RNA, recommended by the manufacturer; black spots) and 2 μl of total RNA (gray spots). Each spot is an analyzed sample for each volume condition (5 μl; 2 μl). For graphs **(A,E)**, the line connecting the points shows the paired results obtained from the same sample analyzed by loading the two different volumes. Cq = 46 denotes no amplification (No Cq). **(A)** Paired quantification cycle (Cq) analysis for RNase P probe of each sample assessed. **(B)** Cq means value (mean ± SD) for the RNase P probe amplification. RNase P probe amplification curves loading **(C)** 5 μl and **(D)** 2 μl of total RNA. **(E)** Paired Cq analysis for RdRp probe of each sample assessed. **(F)** Cq means value (mean ± SD) for the RdRp probe amplification. The broken line indicates the Cq cut-off value (Cq ≤ 39) recommended by the manufacturer. RdRp probe amplification curves loading **(G)** 5 μl and **(H)** 2 μl of total RNA. For statistical analysis, paired two-sided Student *T*-test was applied (*n* = 90 NPS blind selected samples, 24 positives for SARS-CoV-2). **p < 0.01; ****p < 0.0001.

Concerning the analysis of the performance of the Roche RT-qPCR kit, variations were determined between the assay parameters, about the volume recommended by the manufacturer (5 μl) and 2 μl of total RNA. These differences were mostly observed in the SARS-CoV-2 RdRp viral probe. Looking at the Cq values for the RNase P probe, 2 μl and 5 μl total RNA showed significant differences between them (Figure 3A), with a slightly lower mean Cq value for 2 μl (18, 22 ± 1.76) than 5 μl of the template (18.63 ± 1.95) (Figure 3B). However, the RFU values for the RNase P probe showed a significant difference between both template volumes (Supplementary Figure 1I), determined by a higher mean fluorescence for the 2 μl (6382 ± 758.4) than 5 μl of total. RNA template (4944 ± 840.2) (Supplementary Figure 1J). RNase P probe amplification curves had higher RFU when 2 μl total RNA was loaded (Figure 3D), compared to 5 μl total RNA (Figure 3C). This result indicates a higher amplification quality when 2 μl of total RNA is loaded, and therefore, better diagnostic quality.

The amplification of the probe for detecting the viral gene RdRp determined that 18 of 24 samples were diagnosed as positive for COVID-19, using 5 μl of total RNA. Instead, 24 samples were diagnosed as positive when 2 μl of RNA was used (Figure 3E). The differences in lower RdRp probe Cq values recorded for the 2 μl total RNA template (29.98 ± 5.95) than the 5 μl total RNA (32.48 ± 8.91) may explain a better performance of the kit for lower amounts of RNA (Figure 3F).

Similar to the amplification observed for the RNase P probe, significantly higher fluorescence was found in the amplification for RdRp with 2 μl compared to 5 μl of total RNA (Supplementary Figure 1K). Therefore, the mean fluorescence for the 2 μl total RNA was higher (1799 ± 376.9) than that found for the 5 μl total RNA samples (1204 ± 538.9) (Supplementary Figure 1L).

The amplification curves of the RdRp probe showed more resolved curves and a higher RFU profile when using 2 μl of RNA (Figure 3H) compared to 5 μl of RNA (Figure 3G), which reflects a higher quality of amplification when 2 μl of total RNA is loaded.

The results of the Roche RT-qPCR kit confirm the higher performance for the detection of SARS-CoV-2 loading 2 μl of total RNA instead of the volume recommended by the manufacturer. Therefore, these analyzes allowed us to determine the best conditions and quality for the diagnosis of COVID-19 using the three kits.

### Efficiency and standard curves of internal control and viral probes

Once the volume of total RNA loaded into the RT-qPCR reaction was standardized to 2 μl for all RT-qPCR kits, an efficiency curve was performed. This curve was made using 10-fold serial dilutions of a reference pool consisting of 10 positive samples whose Cq value was close to 20. This analysis determined the efficiency of the SARS-CoV-2 viral probes and their internal controls. In addition, through this analysis, the Cq cut-off of the probes of each RT-qPCR kit was determined, understood as the average of the Cq values of the last dilution that showed amplification. Then, a standard curve was performed for each probe using serial 10-fold dilutions of the positive control from its respective RT-qPCR kit to quantify the number of genome copies/μl detected by each RT-qPCR kit.

**Figure 4:**
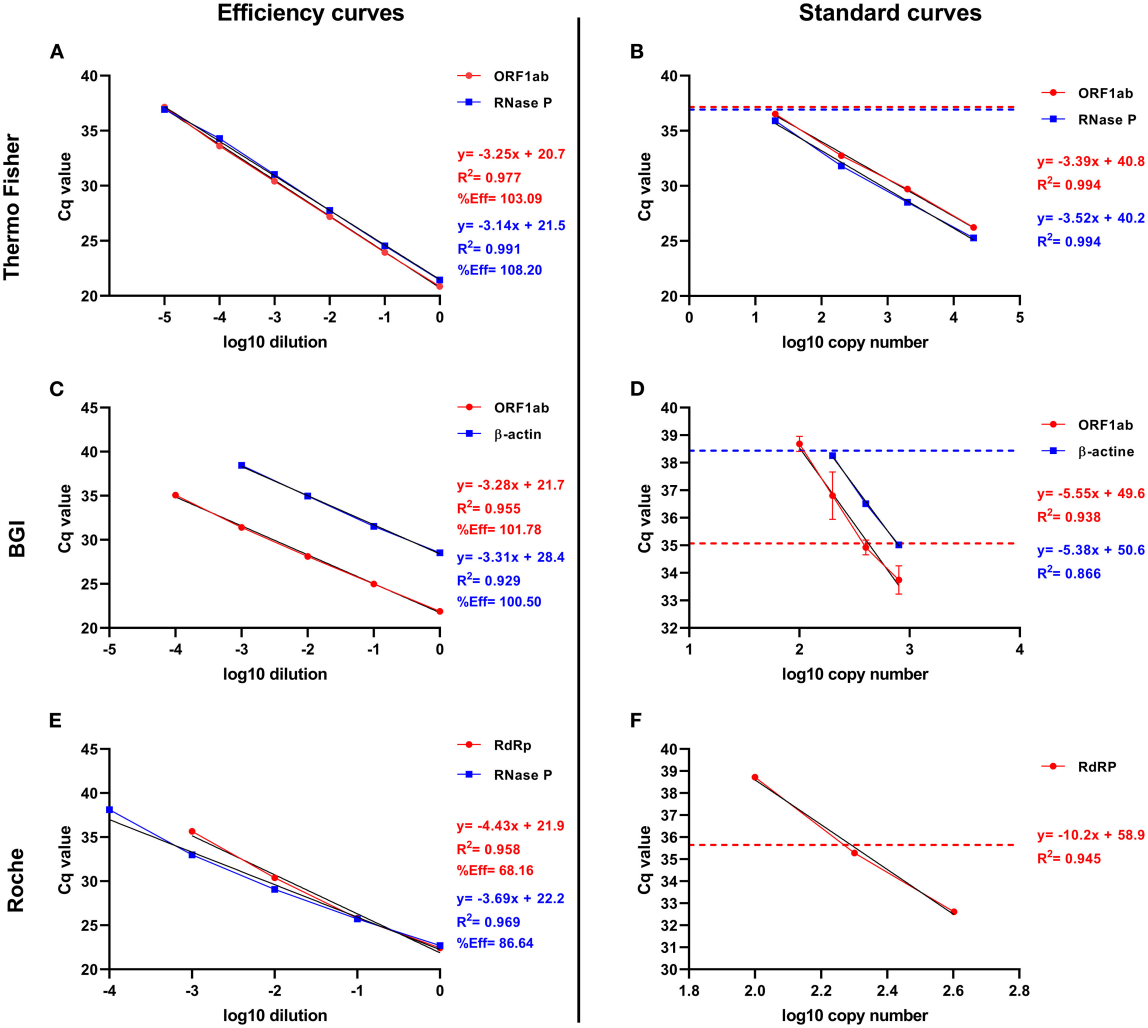
Efficiency and standard amplification curves to determine probe efficiency, Cq cut-off, and genome copies number for the Thermo Fisher, BGI, and Roche RT-qPCR kits. The left and right columns show the efficiency and the standard curves, respectively. The graphs represented the information about the viral and IC probes with red and blue colors, respectively. **(A)** Efficiency curve for ORF1ab and RNase P probes from Thermo Fisher RT-qPCR kit. **(B)** Standard curve for ORF1ab and RNase P probes from Thermo Fisher RT-qPCR kit. **(C)** Efficiency curve for ORF1ab and β-actin probes from BGI RT-qPCR kit. **(D)** Standard curve for ORF1ab and β-actin probes from BGI RT-qPCR kit. **(F)** Efficiency curve for RdRp probe from Roche RT-qPCR kit combined with RNase P probe from Thermo Fisher RT-qPCR kit. **(E)** Standard curve for RdRp probe (Roche RT-qPCR kit). The standard curve for the IC probe from the Roche kit was not plotted because the RNase P probe from the Thermo Fisher kit was used in the Roche RT-qPCR reaction, and the Roche positive control is recognized by the Equine Arteritis Virus (EAV) probe. The graphs represent the linear equation (y = a+bx, b = slope, and a = y-intercept), R-squared (R^2^), and the percentage probe efficiency (%Eff). The broken line indicates the value at which the Cq cut-off was set for each probe assessed for Thermo Fisher (CqRNaseP = 36.92; Cq_ORF1ab_ = 37.15), BGI (Cq_β-actin_ = 38.44; Cq_ORF1ab_ = 35.07), and Roche RT-qPCR kit (Cq_RdRp_ = 35.65).

For the Thermo Fisher RT-qPCR kit, the efficiency of the ORF1ab probe was 103.09%. The Cq cut-off was 37.15 (Figure 4A), corresponding to 5.97 copies/μl (Figure 4B). While the efficiency of the RNase P probe was 108.20% with a Cq cut-off of 36.92 (Figure 4A), corresponding to 4.27 copies/μl (Figure 4B). In the case of the BGI RT-qPCR kit, the efficiency of the ORF1ab probe was 101.78%, with a Cq cut-off of 35.07 (Figure 4C), corresponding to 207.49 copies/μl (Figure 4D). The efficiency of the β-actin probe was 100.50%, with a Cq cut-off of 38.44 (Figure 4C), corresponding to 91.03 copies/μl (Figure 4D). Finally, for the Roche RT-qPCR kit, the efficiency of the RdRp probe was 68.16%. The Cq cut-off was 35.65 (Figure 4E), corresponding to 95.14 copies/μl (Figure 4F). While the efficiency of the RNase P probe was 86.64%, and the Cq cut-off of 38.10 (Figure 4E), whose corresponding number of copies/μl could not be determined.

### Sensitivity comparison between the three RT-qPCR kits

**Figure 5:**
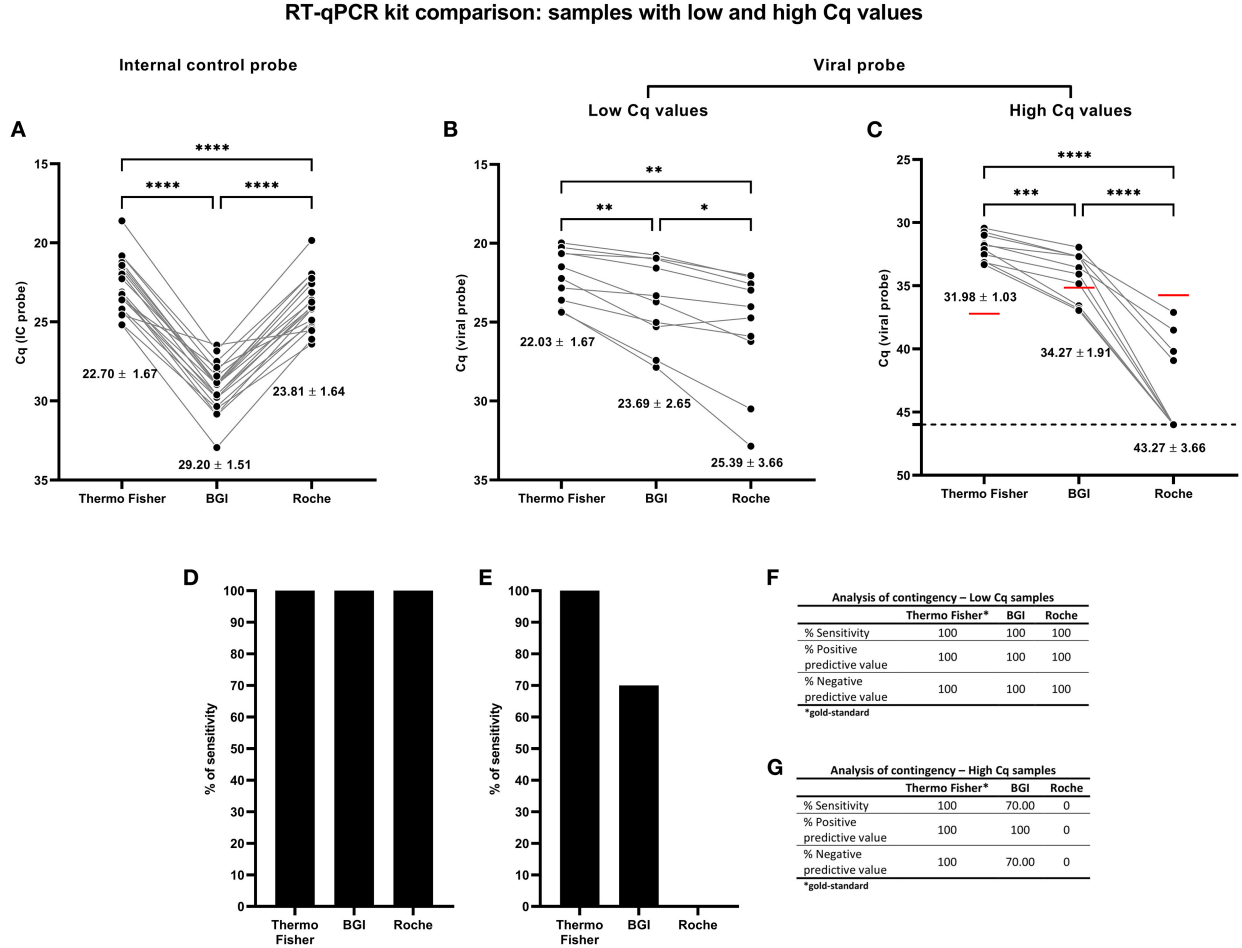
Comparative analysis for detecting SARS-CoV-2 from NPS samples with low (Cq < 30) and high (Cq > 30) viral probe Cq values using the three RT-qPCR kits. The comparison was made from the same NPS sample loading the optimized volume of total RNA extracted (2 μl). Each spot for each RT-qPCR kit is a different analyzed sample. For graphs **(A–C)**, the lines connecting the points indicated the paired result obtained from the same sample assessed by the different RT-qPCR kits; the numbers below each group of points represent the mean Cq value and the standard deviation (mean ± SD). Cq = 46 denotes no amplification (No Cq) and is represented with a broken line. **(A)** Paired Cq analysis for the IC probe (RNase P or β-actin) amplification values obtained by RT-qPCR for each sample assessed. Paired Cq analysis for the SARS-CoV-2 viral probe (ORF1ab or RdRp) amplification values obtained by RT-qPCR for samples with **(B)** low Cq value (Cq value < 30, high viral load) and **(C)** high Cq value (Cq value > 30, low viral load). In graph **(C)**, the horizontal red lines indicate the Cq cut-off determined for the viral probe (Figure 4) of each RT-qPCR kit respectively. Comparison of RT-qPCR kits sensitivity percentage assessed for samples with **(D)** low and **(E)** high viral probe Cq values for all three RT-qPCR kits. Contingency analysis for all three RT-qPCR kits for samples with **(F)** low and **(G)** high viral probe Cq values for all three RT-qPCR kits. For statistical analysis, paired two-way ANOVA was applied (*n* = 10 NPS samples with Cq value < 30; *n* = 10 NPS samples with Cq value > 30). *p < 0.05; **p < 0.01; ***p < 0.001; ****p < 0.0001.

The sensitivity comparison between the three different RT-qPCR kits was performed using SARS-CoV-2 (wild-type) ancestral samples. Twenty samples of NPSs positive for SARS-CoV-2 were used; ten with low Cq value (Cq < 30, high viral load) and ten positive NPS samples with high Cq value (Cq > 30, low viral load) previously diagnosed with the Thermo Fisher RT-qPCR kit (gold standard kit). For all twenty samples, IC probe amplification showed significant differences in Cq values between matched NPSs for RT-qPCR kits (Figure 5A). The IC probe amplification Cq was most significant for the Thermo Fisher kit (22.70 ± 1.67), followed by the Roche kit (23.81 ± 1.64) and the BGI kit (29.20 ± 1.51) (Figure 5A). Then, the detection of the SARS-CoV-2 viral genes was determined. Comparing the samples evaluated for each kit, all samples with low and high viral probe Cq values showed significant differences in the best assay conditions previously analyzed. In samples with low Cq values of the viral probe Cq < 30 (high viral load), mean Cq values were slightly different between kits, with 22.03 ± 1.67 for Thermo Fisher; 23.69 ± 2.65 for BGI and 25.39 ± 3.66 for Roche (Figure 5B). However, for samples with high viral probe Cq values (Cq > 30, low viral load), there were wider differences between kits, with 31.98 ± 1.03 for Thermo Fisher; 34.27 ± 1.91 for BGI and 43.27 ± 3.66 for Roche (Figure 5C). These results have an impact on the RT-qPCR kit sensitivity. In the samples with a low viral probe Cq value, RFU values were also affected, which also indicates a difference on the amplification curve (Supplementary Figure 2), although fluorescence is not directly related to the sensitivity of the different kits evaluated.

The three RT-qPCR kits showed 100% sensitivity for high viral load (Cq < 30) (Figure 5D). By contrast, for the samples with a high Cq value (low viral load), the sensitivity was 100% for Thermo Fisher and 70% for BGI, meanwhile the sensitivity was 0% for Roche (Figure 5E). In this sense, all the Cq values of the samples evaluated by Roche were outside the Cq cut-off of the kit or showed no amplification (No Cq) (Figure 5C). For the three RT-qPCR kits, the contingency analysis showed 100% positive and negative predictive values for samples with low Cq values (Cq < 30) for the viral probe (Figure 5F). On the other hand, for samples with high Cq values (Cq > 30), the contingency analysis showed 100, 70 and 0% positive predictive values, for the Thermo Fisher, BGI and Roche kits, respectively (Figure 5G). These results suggest performance and sensitivity of 100% for the three kits in samples with high viral load.

### Sensitivity comparison between the three RT-qPCR kits against variants of SARS-CoV-2

**Figure 6:**
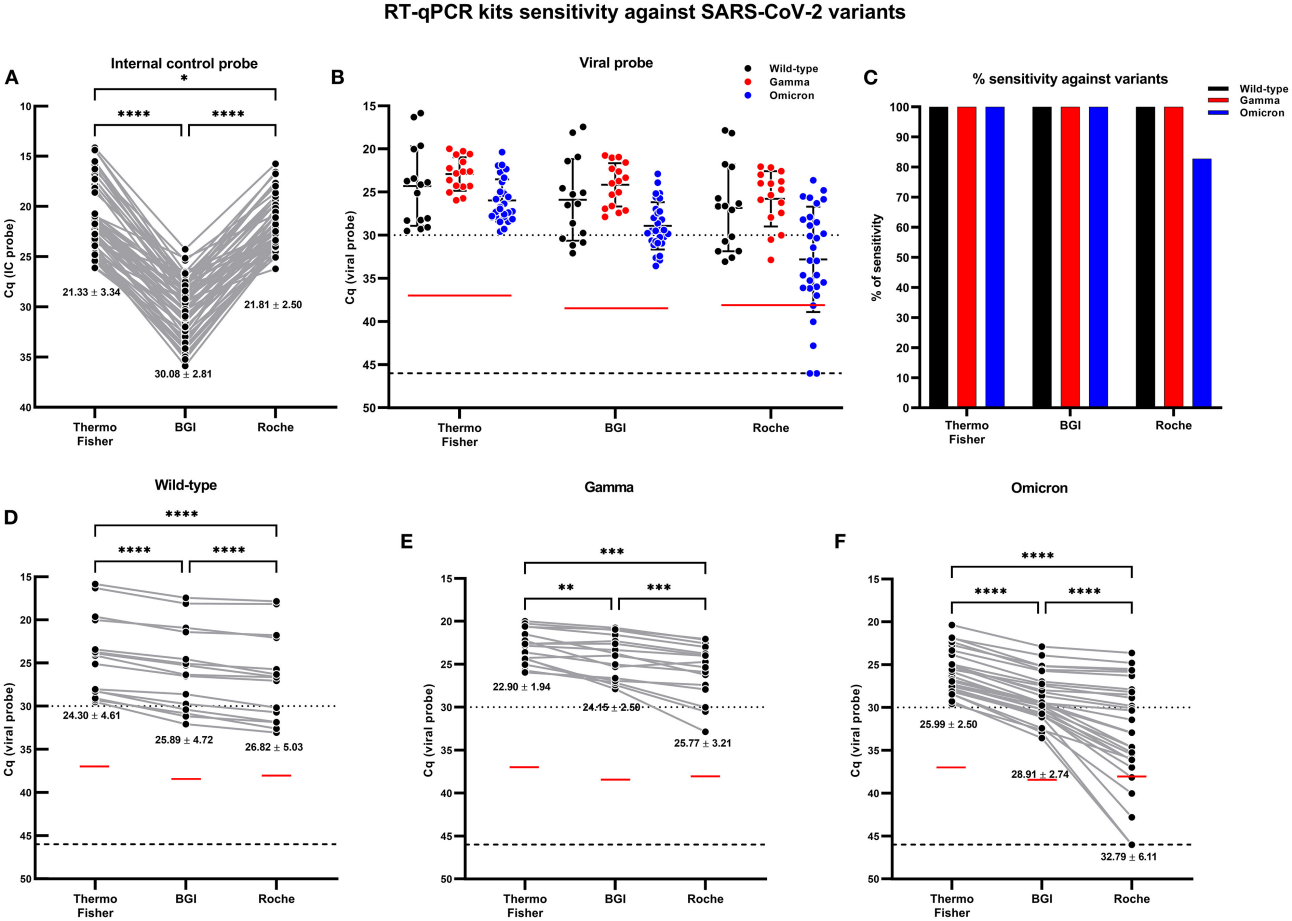
Comparative analysis of SARS-CoV-2 variants detection from NPS samples using the three RT-qPCR kits. The comparison was made from the same NPS sample loading the optimized volume of total RNA extracted (2 μl). Each spot for each RT-qPCR kit is a different analyzed sample. For graphs **(A,D–F)**, the lines connecting the points indicated the paired result obtained from the same sample assessed by the different RT-qPCR kits; the numbers below each group of points represent the mean Cq value, and the standard deviation (mean ± SD). Cq = 46 denotes no amplification (No Cq) and is represented with a broken line. The horizontal red lines on graphs **(B,D–F)**, indicate the Cq cut-off determined for the viral probe (Figure 4) of each RT-qPCR kit, respectively. **(A)** Paired Cq analysis for the IC probe (RNase P or β-actin) amplification values obtained by RT-qPCR for each sample assessed. **(B)** Comparative distribution of Cq values for SARS-CoV-2 variant samples diagnosed by each RT-qPCR kit. **(C)** RT-qPCR kit sensitivity percentage of RT-qPCR kits against SARS-CoV-2 variants. Paired Cq analysis for the SARS-CoV-2 viral probe (ORF1ab or RdRp) amplification values obtained by RT-qPCR for each sample assessed corresponding to **(D)** wild-type strain SARS-CoV-2, **(E)** Gamma variant, and **(F)** Omicron variant. For statistical analysis, paired two-way ANOVA was applied (60 samples were selected, of which 15, 16, and 29 samples correspond to the wild-type strain, the Gamma variant, and the Omicron variant, respectively). *p < 0.05; **p < 0.01; ***p < 0.001; ****p < 0.0001.

After evaluating the sensitivity of the three RT-qPCR kits using ancestral SARS-CoV-2 samples, the sensitivity of the three kits was analyzed using NPSs corresponding to SARS-CoV-2 variants (Gamma and Omicron), which yielded a Cq value < 30 when diagnosed with the Thermo Fisher RT-qPCR kit. Firstly, the Cq values for IC were compared between the different kits for the 60 samples analyzed (between the ancestor strain, Gamma variant, and Omicron). The Cq mean value of Thermo Fisher and Roche kits showed a slight difference. The BGI kit showed the highest Cq values for IC detection. Mean Cq values were 21.33 ± 3.34, 30.08 ± 2.81, and 28.81 ± 2.50 for the Thermo Fisher, BGI, and Roche kits, respectively (Figure 6A).

When wild-type, Gamma, and Omicron samples were tested, the Cq values obtained for the viral probe were greater in the Roche and BGI kits compared to the result obtained with the Thermo Fisher kit, with higher values in Omicron variant for Roche (Figure 6B). All three kits showed 100% sensitivity for detection of the Gamma variant; the Thermo Fisher and BGI kits also showed 100% sensitivity for the Omicron variant (determined by mutations K417T, del69-70, and N501Y), while the Roche kit showed a sensitivity of 82.7% for the detection of Omicron in NPSs with Cq < 30 (Figure 6C). When performing a pairwise comparison between the results obtained with the three kits for each variant, the increase in the Cq value for the viral probe is more evident. For the ancestral virus (Wild type), differences in viral Cq values were observed, with mean Cq values for viral probe of 24.30 ± 4.61, 25.89 ± 4.72, and 26.82 ± 5.03 for Thermo Fisher, BGI, and Roche, respectively (Figure 6D). In the case of the samples corresponding to the Gamma variant, the kits showed significant differences between them, with an increase in Cq values for the Roche and BGI kit. The mean Cq values were 22.90 ± 1.94, 24.45 ± 2.50, and 25.77 ± 3.21 for Thermo Fisher, BGI, and Roche, respectively (Figure 6E).

Finally, the Cq values against the analysis of the Omicron variant were sometimes higher than the detection limit for the Roche kit (Figure 6F). In this case, five samples showed Cq values greater than the Cq cut-off (38.10) previously determined for this kit or showed no amplification (No Cq). This implies that the Roche kit showed a sensitivity percentage of 82.7% against the Omicron variant in samples with a high viral load.

## Discussion

Previous works have analyzed the detection capacity of different commercial RT-qPCR kits used to diagnose COVID-19 in other countries (12, 13, 19, 20). For example, Lu et al. (21) compared and analyzed the performance of Sansure and BioGerm RT-qPCR kits, widely used in Liuzhou People's Hospital in Guangxi, China, with a sensitivity of 83.3 and 94.4%, respectively. Eberle et al. (22) compared nine RT-qPCR kits used in viral diagnosis in Bavaria, Germany. Most of them reached a sensitivity between 90-100%, while two kits reported 49% (Gerbion GmbH & Co KG) and 62% (Wells Bio, Inc) sensitivity with the highest number of false negatives. Another report, carried out in the Republic of Serbia, concluded that the three most used RT-qPCR kits had a detection sensitivity of 91% up to almost 96% (23).

In the Latin American scenario, little and lower quality of RT-qPCR kits have been reported in relation to other developed countries outside the region (11). In this sense, the MaxCov19 from TAAG Genetic kit used in Chile during the pandemic showed a sensitivity of up to 65% (8), while the AccuPower SARS-CoV-2 Real Time RT-PCR kit used in Ecuador in the first months of the pandemic, showed a sensitivity of 78.9% (24), a value of sensitivity close but below to the World Health Organization recommendation (≥ 80% and desirable ≥ 90%) (25). In Colombia, nine different RT-qPCR kits were evaluated, showing a sensitivity that varied between 87.76 and 100%, suggesting a good diagnosis in the country (7). Furthermore, none of these previous studies have differentiated the variant of SARS-CoV-2 analyzed, which may be responsible for the different sensitivities reported in the above-mentioned studies.

Our study is the first comparative analysis of the TaqMan 2019-nCoV Assay Kit v1 (Thermo Fisher), the Real-Time Fluorescent RT-PCR Kit for Detecting SARS-CoV-2 (BGI), and the LightCycler^®^ Multiplex RNA Virus Master (Roche) commercial RT-qPCR kits used for massive testing of the population of Santiago, Chile, and authorized for use at the national level by the health authorities. This also includes the sensitivity analysis against the Omicron variant compared to other variants, such as Gamma, which have high transmissibility (26) and has caused numerous deaths (27), respectively.

Our study revealed significant differences in Cq amplification values for the viral probe among the three RT-qPCR kits tested using 2 μl of RNA instead of the manufacturer's recommended volume of RNA. The effect of using less RNA was also manifested in an increase in RFU as an indicator of the quality of the amplification. Thus, the curves showed a better resolution of the amplification curves and a steeper slope. The improve on these amplification parameters could be explained by the dilution or lower concentration of unknown inhibitors present in the reaction, carried over from the RNA extraction step, as previously distinguished (28, 29). Anyway, the sensitivity of a RT-qPCR kit is directly related to viral Cq values but not the RFU of the assay.

For efficiency analysis of an RT-qPCR assay we followed the recommendations made by Svec et al. (30) and Bustin et al. (31). Thus, the correct calculation of efficiency includes a standard curve with three replicates for each concentration, same analytical instrument for measurement, low concentrations of RNA, and more than three data points to obtain the curve (30, 31). The efficiency analysis results could explain the Roche kit's low sensitivity against samples with low viral load. These adjustments and standardization of parameters can reduce the chances of false-negative diagnoses and improve control of COVID-19, in the same way as previously reported in the standardization of efficiency curves of other commercial RT-qPCR kits (8). Another relevant issue in RT-qPCR for diagnostic purposes is the choice of the Cq cut-off with which a sample is validated as positive or negative. The choice of Cq cut-off cannot be arbitrary, since choosing a very low value produces false negative results, and a very high value produces unreliable positive results (31, 32). Although the kit manufacturer establishes the Cq cut-off, it is necessary to base it on empirical evidence by drawing up calibration curves under laboratory working conditions and comparative analyzes of previously diagnosed samples (8).

Our findings suggest the use of the Thermo Fisher kit as a gold standard for SARS-CoV-2 diagnosis. This idea agrees with the evidence reported by Farfán et al. (33). Other reports describe the performance of in-house RNA SARS-CoV-2 extraction protocols, validating their results with the same Thermo Fisher kit used in our study (33). In addition, others point out its compatibility to detect SARS-CoV-2 in nasopharyngeal samples without prior RNA extraction (34). Taken together, these results corroborate the good performance and sensitivity of the viral detection by Thermo Fisher kit.

Despite the improvements in the amplification parameters after modifying the volume of loaded RNA, the sensitivity of the BGI and Roche RT-qPCR kits were still lower compared to Thermo Fisher kit. While the BGI kit has shown a sensitivity of ≥ 95% in other studies (13), we obtained a sensitivity of 100% in samples with high viral loads (Cq < 30). However, in our study the Roche kit dropped to 0% sensitivity compared to Thermo Fisher when analyzing NPSs with low viral loads (Cq > 30). However, studies indicate that Roche performs well enough to detect positive cases compared to other commercial RT-qPCR kits, such as Cepheid and Certest Biotec SL (35). These antecedents reinforce the idea to standardize the RT-qPCR kits for the laboratory working conditions (8).

The three kits had a sensitivity of 100% in samples with high viral load (Cq < 30). However, marked differences arise in our study in samples with low viral load (Cq > 30), where the sensitivity of the Roche kit tends to be 0% with the sample size analyzed (*n* = 10 samples with low viral loads). These results may be due to the low efficiency of the viral probe in the Roche kit, which barely reached 68% under our experimental conditions, where other studies recommend at least 90% efficiency (30). The RT-qPCR reactions with low efficiencies require more cycles to exceed the quantification threshold (36), which reflects a low or late amplification curve for viral/endogenous targets. This sensitivity (0% in Roche kit) could improve by increasing the number of samples analyzed.

An important fact in the development of the SARS-CoV-2 pandemic has been the emergence of genetic variants. The low-fidelity RNA-dependent RNA polymerase (RdRp) of SARS-CoV-2 is responsible for its high adaptive mutation rate which has allowed the appearance of several variants of this virus throughout the pandemic. In the Omicron variant, the acquired mutations have increased its infectivity, the risk of reinfections, and immune escape compared to the ancestral strain (37). According to the Food and Drug Administration (FDA), another consequence of the emergence of SARS-CoV-2 variants could be reduce the sensitivity of some RT-qPCR tests (38). From this concern arise the advice to use more than one target viral probe to increase detection capacity (39), and the need to analyze routinely the performance of commercial RT-qPCR kits used locally in the diagnosis of COVID-19.

Interestingly, the Roche kit shows 82.76% detection of the Omicron variant, while the BGI and Thermo Fisher kits showed 100%; Roche has sufficient sensitivity according to the WHO recommendations. In addition, the analyzes carried out by the manufacturer reported 100% sensitivity for the Omicron variant, so its use is still recommended for low viral loads (Cq < 30) of this variant (40). The Omicron variant and its subvariants were not differentiated for the analysis in this work.

In summary, this study is the first to analyze and compare the sensitivity and performance of RT-qPCR kits widely used in Chile during the COVID-19 pandemic. Our study reveals differences between the Thermo Fisher, BGI, and Roche RT-qPCR kit for SARS-CoV-2 diagnosis, including the detection of Omicron, the currently predominant variant globally (41). The choice of a kit with higher sensitivity means fewer false negatives and better control of current and, prospectively, future viral infectious diseases diagnosed by RT-qPCR.

## Data availability statement

The raw data supporting the conclusions of this article will be made available by the authors, without undue reservation.

## Ethics statement

The studies involving human participants were reviewed and approved by the Ethical Committee of the University of Santiago of Chile (No. 226/2021) and the Scientific Ethical Committee of the Central Metropolitan Health Service, Ministry of Health, Government of Chile (No. 370/2021). Written informed consent for participation was not required for this study in accordance with the national legislation and the institutional requirements.

## Author contributions

CA-C, FER-L, and AMS made the conceptualization of the study. DV, CA-C, FER-L, and AMS supervised the study. DV and CA-C were in charge of all the logistics of coordination and reception of samples from the central metropolitan health service. CB-A, AM-T, and FH were in charge of the logistic of sampling processing and RNA extractions. ÁS, RL, JA, CM-F, and JC received the RNA extracted and performed the RT-qPCR reactions. ÁS, RL, EV-V, FER-L, and AMS analyzed the data. ÁS, RL, EV-V, and FER-L made the data representation. ÁS, CB-A, RL, EV-V, AI-M, MI, CA-C, FER-L, and AMS carried out the data processing and interpretation. ÁS, CB-A, RL, EV-V, MI, and FER-L co-wrote the original draft. All authors provided critical feedback, approved the final manuscript, read, and agreed to the published version of the manuscript.

## Funding

The authors thank to the Rapid Assignment of Resources for Research Projects on the Coronavirus (COVID-19) (project number COVID1038; ANID, Government of Chile), Fondecyt regular project numbers 1201664 (MI) and 1211841 (FER-L) (ANID, Government of Chile), Fondecyt iniciación project number 11221308 (EV-V), and DICYT-USACH project number 021943AC (CA-C) grants. The funders had no role in study design, data collection, and analysis, decision to publish, or preparation of the manuscript.

## Conflict of interest

The authors declare that the research was conducted in the absence of any commercial or financial relationships that could be construed as a potential conflict of interest.

## Publisher's note

All claims expressed in this article are solely those of the authors and do not necessarily represent those of their affiliated organizations, or those of the publisher, the editors and the reviewers. Any product that may be evaluated in this article, or claim that may be made by its manufacturer, is not guaranteed or endorsed by the publisher.

## Supplementary material

Supplementary Figure 1 RFU analysis for optimization of RT-qPCR for detecting SARS-CoV-2 from nasopharyngeal swab (NPS) samples using the internal control and viral probes from Thermo Fisher, BGI, and Roche kits. The comparison was made from the same NPS sample loading the recommended volume of extracted RNA (5 or 10 μl of total RNA, recommended by the manufacturer; black spots) and 2 μl of total RNA (gray spots). Each spot is an analyzed sample for each volume condition (5 or 10 μl; 2 μl). For graphs **(A,C,E,G,I,K)**, the line connecting the points shows the paired results obtained from the same sample analyzed by loading the two different volumes. **(A)** Paired RFU analysis for RNase P probe loading 5 and 2 μl of RNA extracted using Thermo Fisher RT-qPCR kit. **(B)** Mean RFU (mean ± SD) for RNase P probe amplification. **(C)** Paired RFU analysis for ORF1ab probe loading 5 and 2 μl of RNA extracted using Thermo Fisher RT-qPCR kit. **(D)** RFU mean (mean ± SD) for ORF1ab probe amplification. **(E)** Paired RFU analysis for β-actin probe loading 5 and 2 μl of RNA extracted using BGI RT-qPCR kit. **(F)** Mean RFU (mean ± SD) for β-actin probe amplification. **(G)** Paired RFU analysis for ORF1ab probe loading 5 and 2 μl of RNA extracted using BGI RT-qPCR kit. **(H)** RFU mean (mean ± SD) for ORF1ab probe amplification. **(I)** Paired RFU analysis for RNase P probe loading 5 and 2 μl of RNA extracted using Roche RT-qPCR kit. **(J)** Mean RFU (mean ± SD) for RNase P probe amplification. **(K)** Paired RFU analysis for RdRp probe loading 5 and 2 μl of RNA extracted using Roche RT-qPCR kit. **(L)** RFU mean (mean ± SD) for RdRp probe amplification. For statistical analysis, paired two-sided Student *T*-test was applied (For Thermo Fisher kit, *n* = 91 NPS blind selected samples, 20 of these positive for SARS-CoV-2; for BGI kit, *n* = 70 NPS blind selected samples, 42 of these positive for SARS-CoV-2; and for Roche kit, *n* = 90 NPS blind selected samples, 24 of these positive for SARS-CoV-2;). ^*^*p* < 0.05; ^**^*p* < 0.01; ^***^*p* < 0.001; ^****^*p* < 0.0001.

Supplementary Figure 2 Comparative RFU analysis for detecting SARS-CoV-2 from NPS samples with low (Cq < 30) and high (Cq > 30) viral probe Cq values using the three RT-qPCR kits. The comparison was made from the same NPS sample loading the optimized volume of total RNA extracted (2 μl). Each spot for each RT-qPCR kit is a different analyzed sample. For graphs **(A–C)**, the lines connecting the points indicated the paired result obtained from the same sample assessed by the different RT-qPCR kits; the numbers below each group of points represent the mean Cq value and the standard deviation (mean ± SD). **(A)** Paired RFU analysis for the IC probe (RNase P or β-actin) amplification values obtained by RT-qPCR for each sample assessed. Paired RFU analysis for the SARS-CoV-2 viral probe (ORF1ab or RdRp) amplification values obtained by RT-qPCR for samples with **(B)** low Cq value (Cq value < 30, high viral load), and **(C)** high Cq value (Cq value > 30, low viral load. For statistical analysis, paired two-way ANOVA was applied (*n* = 10 NPS samples with Cq value < 30; *n* = 10 NPS samples with Cq value > 30). ^*^*p* < 0.05; ^**^*p* < 0.01; ^***^*p* < 0.001; ^****^*p* < 0.0001.
