# Supplementary figures and images for: Evaluation and comparison of the sensitivity of three commercial RT-qPCR kits used for the detection of SARS-CoV-2 in Santiago, Chile
Source: Front Public Health. 2022 Nov 28;10:1010336. doi: 10.3389/fpubh.2022.1010336 (PMC9742446; doi:10.3389/fpubh.2022.1010336)

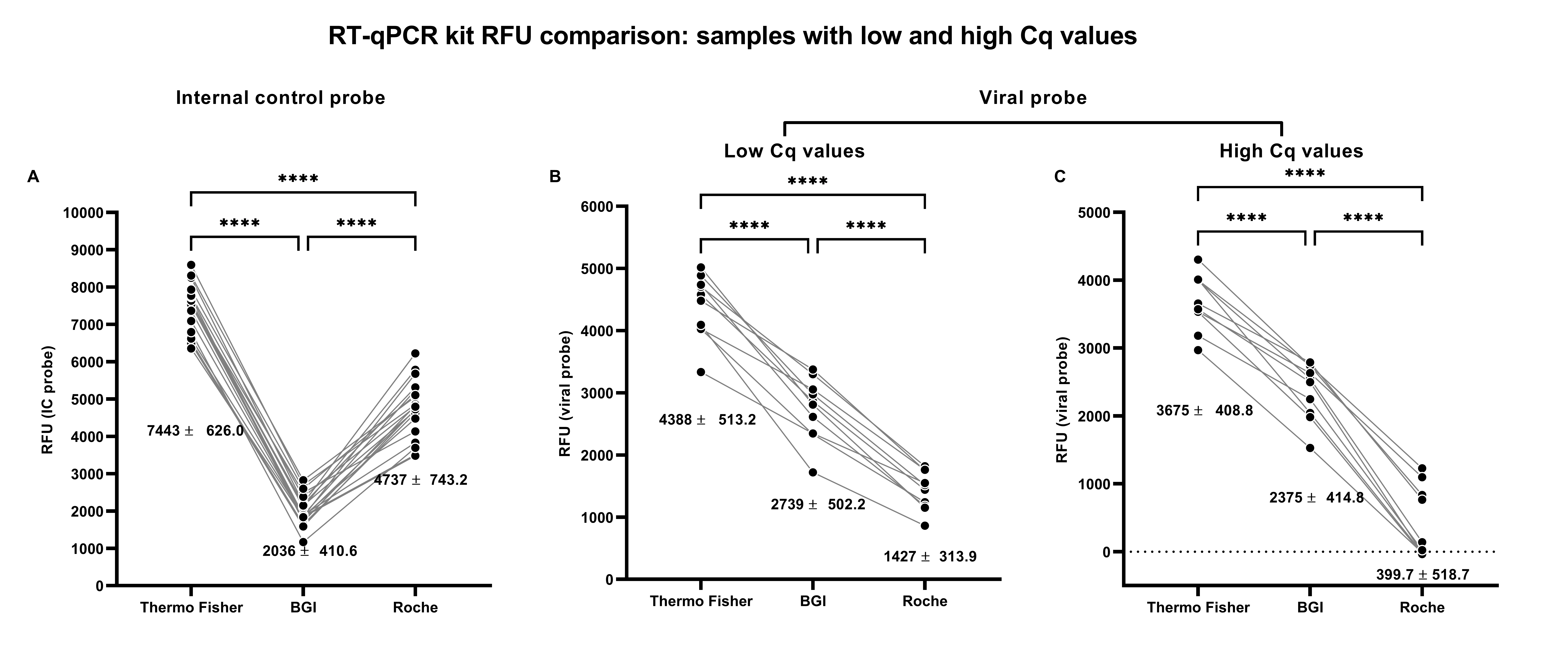

Supplement: Supplementary Figure 2 — Comparative RFU analysis for detecting SARS-CoV-2 from NPS samples with low (Cq < 30) and high (Cq > 30) viral probe Cq values using the three RT-qPCR kits. The comparison was made from the same NPS sample loading the optimized volume of total RNA extracted (2 μl). Each spot for each RT-qPCR kit is a different analyzed sample. For graphs (A–C), the lines connecting the points indicated the paired result obtained from the same sample assessed by the different RT-qPCR kits; the numbers below each group of points represent the mean Cq value and the standard deviation (mean ± SD). (A) Paired RFU analysis for the IC probe (RNase P or β-actin) amplification values obtained by RT-qPCR for each sample assessed. Paired RFU analysis for the SARS-CoV-2 viral probe (ORF1ab or RdRp) amplification values obtained by RT-qPCR for samples with (B) low Cq value (Cq value < 30, high viral load), and (C) high Cq value (Cq value > 30, low viral load. For statistical analysis, paired two-way ANOVA was applied (n = 10 NPS samples with Cq value < 30; n = 10 NPS samples with Cq value > 30). *p < 0.05; **p < 0.01; ***p < 0.001; ****p < 0.0001. [file Image_2.TIF]
